# Chitosan Nanocomposite Coatings Containing Chemically Resistant ZnO–SnO_x_ Core–shell Nanoparticles for Photocatalytic Antifouling

**DOI:** 10.3390/ijms22094513

**Published:** 2021-04-26

**Authors:** Santosh Kumar, Fei Ye, Babak Mazinani, Sergey Dobretsov, Joydeep Dutta

**Affiliations:** 1 Department of Food Engineering and Technology, Central Institute of Technology, Kokrajhar 783370, India; s.kumar@cit.ac.in; 2 Functional Materials Group, Department of Applied Physics, School of Engineering Sciences, KTH Royal Institute of Technology, Hannes Alfvéns väg 12, 114 19 Stockholm, Sweden; 3 Department of Materials Engineering, Malayer University, Malayer 65719-95863, Iran; b.mazinany@gmail.com; 4 Department of Marine Science, Sultan Qaboos University, PO Box 34, Al Khoud, Muscat 123, Oman; sergey_dobretsov@yahoo.com; 5 Center of Excellence in Marine Biotechnology, Sultan Qaboos University, PO Box 50, Al Khoud, Muscat 123, Oman

**Keywords:** chitosan, ZnO, nanocomposite, chemically resistant, photocatalytic, antifouling

## Abstract

Functional nanocomposites with biopolymers and zinc oxide (ZnO) nanoparticles is an emerging application of photocatalysis in antifouling coatings. The reduced chemical stability of ZnO in the acidic media in which chitosan is soluble affects the performance of chitosan nanocomposites in antifouling applications. In this study, a thin shell of amorphous tin dioxide (SnO_x_) was grown on the surface of ZnO to form ZnO–SnO_x_ core–shell nanoparticles that improved the chemical stability of the photocatalyst nanoparticles, as examined at pH 3 and 6. The photocatalytic activity of ZnO–SnO_x_ in the degradation of methylene blue (MB) dye under visible light showed a higher efficiency than that of ZnO nanoparticles due to the passivation of electronic defects. Chitosan-based antifouling coatings with varying percentages of ZnO or ZnO–SnO_x_ nanoparticles, with or without the glutaraldehyde (GA) crosslinking of chitosan, were developed and studied. The incorporation of photocatalysts into the chitosan matrix enhanced the thermal stability of the coatings. Through a mesocosm study using running natural seawater, it was found that chitosan/ZnO–SnO_x_/GA coatings enabled better inhibition of bacterial growth compared to chitosan coatings alone. This study demonstrates the antifouling potential of chitosan nanocomposite coatings containing core–shell nanoparticles as an effective solution for the prevention of biofouling.

## 1. Introduction

Biofouling is the undesirable growth of attached organisms on marine installations [1], which is known to incur billions of dollars in shipping and maintenance costs [2]. To prevent biofouling, industries use antifouling paints containing toxic organics, such as isothiazolone, or inorganic biocides such as copper (Cu) and copper pyruvate, which leach out of the coatings and kill the fouling organisms [3]. However, these toxic biocides also kill non-target marine organisms and are known to accumulate in marine environments [3,4]. Although some non-toxic antifouling coatings are commercially available, they are expensive and not as effective as traditional biocides [5,6]. Thus, there is an urgent need to develop broadly effective, low- or non-toxic antifouling solutions.

The biopolymer chitosan (CH) may offer an ideal antifouling coating solution owing to its broad-spectrum antibacterial, antifungal and anti-algal properties, along with excellent film-forming properties [7,8,9]. Chitosan, a deacetylated form of chitin (a polymer of *N*-acetylglucosamine), is the second most abundant polysaccharide after cellulose on the planet, and is a low-cost, non-toxic, biocompatible material. Furthermore, chitosan is part of a green chemistry strategy as it is mainly extracted from marine shellfish wastes (e.g., exoskeleton of shrimps or crabs) by a very simple and economic protocol [7]. Due to the presence of reactive amino groups, chitosan binds strongly with negatively charged surfaces and easily forms films, coatings and complexes with polyanions. The amine groups are also responsible for the high hydrophilicity of chitosan in an acidic medium [10]. The crosslinking of chitosan molecules via the reaction of primary amine groups in chitosan with aldehyde groups from the crosslinker glutaraldehyde (GA) lead to increased tensile strength, reduced hydrophilicity, and enhanced chemical resistance. Apart from the newly formed imine group, a number of amine groups still remain in the crosslinked chitosan matrix, allowing interaction with the surroundings [11].

Chitosan has antimicrobial properties, primarily due to interactions between the positively charged amine groups of chitosan and the negatively charged microbial cell membranes, leading to the leakage of cellular constituents and consequent cell death [7]. However, further improvements in antimicrobial and other properties are desirable in order to develop practically applicable antifouling coatings [12]. Metal oxide nanoparticles, especially zinc oxide (ZnO), have attracted particular interest for their broad-spectrum antimicrobial (i.e., antibacterial, antiviral, and antifungal) activity, with minimal or no adverse effects on mammalian cells [13,14]. Furthermore, ZnO nanoparticles are listed as a generally recognized as safe (GRAS) material by the US Food and Drug Administration (FDA) [15]. The incorporation of ZnO nanoparticles into chitosan coatings/films has been demonstrated to yield improved antimicrobial activity, mechanical strength, thermal stability, and UV blocking properties [16,17,18,19]. Although recent studies revealed that chitosan–ZnO nanoparticle-based composites have promising potential for application in antifouling coatings [14,20,21], ZnO nanoparticles are chemically unstable under acidic pH (lower than 7) conditions, resulting in Zn^2+^ ions being released from the ZnO nanoparticles [22]. On the other hand, chitosan is only soluble in water under acidic conditions, since the protonation constant (pKa) of chitosan is generally between 6 and 6.5 [23], and the crosslinking of chitosan is most effective at a pH around 4 [24].

Thus, to overcome the stability problem of ZnO in acidic media, in which chitosan is soluble, in this work, a shell of amorphous tin dioxide (SnO_x_) was grown on the surface of ZnO to form ZnO–SnO_x_ core–shell nanoparticles by a simple hydrothermal method. The motivation of choosing SnO_x_ as a coating material on ZnO lies in the chemical stability of SnO_x_ in a broad range of pH values, its wide bandgap (3.6 eV) with low n-type resistivity, and its good transparency that will not hinder, but will instead improve, the photoactivity of ZnO [25]. Previously, ZnO–SnO_x_ core–shell nanorods or nanoparticles have been reported in such applications as optical instruments [26,27] and gas-sensing devices [28,29]. Chitosan–inorganic photocatalyst composites for the visible light-driven decontamination of wastewater, and for antimicrobial or antifouling applications using noble metals, metal oxides or metal chalcogenides in chitosan as support, are also reported [30,31,32]. However, the investigation of ZnO–SnO_x_ core–shell nanostructures as chemically resistant photocatalytic anti-fouling materials has not been carried out. Herein, we incorporate ZnO–SnO_x_ into the chitosan matrix and apply nanocomposite coatings for antifouling applications. We investigated the effects of SnO_x_ coating on the chemical stability of ZnO nanoparticles at acidic pH levels and the photocatalysis of ZnO–SnO_x_ core–shell nanoparticles on the degradation of methylene blue (MB) dye under visible light irradiation. Finally, the antifouling properties of the developed hybrid nanocomposites were tested in an outdoor mesocosm study using the flow of natural seawater.

## 2. Results

### 2.1. Characterization of ZnO–SnO_x_ Core–Shell Nanoparticles

#### 2.1.1. Microstructural Analysis

**Figure 1:**
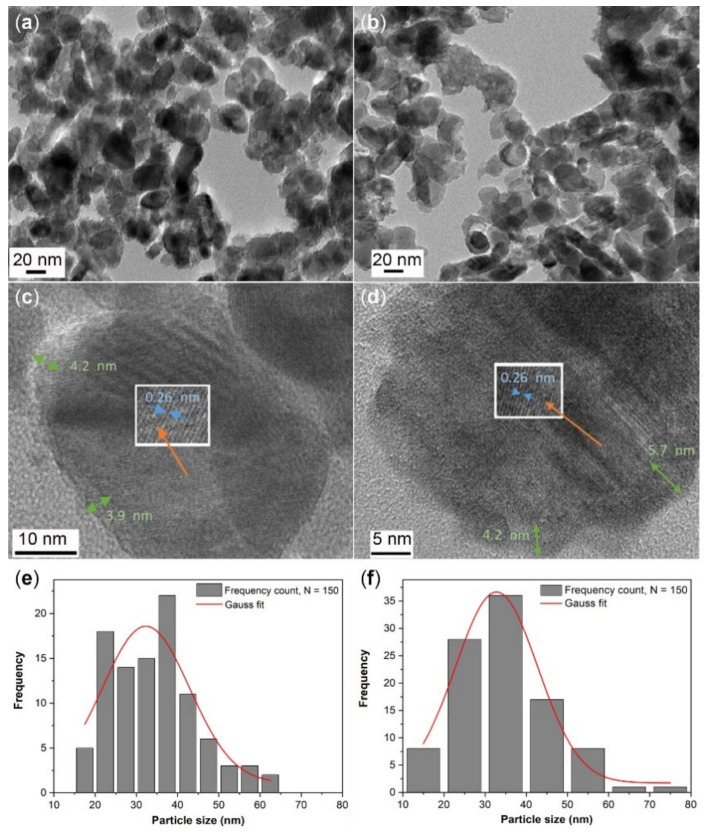
TEM images with low magnification and high magnification of (**a**, **c**) ZnO–SnO_x_ (5 mM) and (**b**,**d**) ZnO–SnO_x_ (10 mM) core–shell nanoparticles. The thickness of the amorphous SnO_x_ coatings is indicated by green arrows and the lattice spacing of ZnO by blue arrows in (**c**) and (**d**); (**e**) and (**f**) show the particle size distribution of ZnO–SnO_x_ (5 mM) and ZnO–SnO_x_ (10 mM) nanoparticles (*n* = 150).

The morphology of ZnO–SnO_x_ core–shell nanoparticles was studied by high-resolution transmission electron microscopy (HRTEM) and the images are shown in Figure 1a–d. An inhomogeneous coating layer of SnO_x_ was observed on the surfaces of the ZnO nanoparticles (Figure 1a,b), where the ZnO nanoparticles were either partially or fully coated. The SnO_x_ coating was found to have an amorphous nature, with a thickness of about 3–4 nm for ZnO–SnO_x_ (5 mM; synthesized using 0.090 g (5 mM) of SnCl_2_·2H_2_O) and 4–6 nm for ZnO–SnO_x_ (10 mM; synthesized using twice the amount of SnCl_2_·2H_2_O precursor) (Figure 1c,d). The lattice fringes of the inner ZnO particles can be clearly observed, and the lattice spacing was about 0.26 nm, which corresponds to the (002) lattice plane of hexagonal ZnO. The particle size distribution of the quasi-spherical particles is summarized in Figure 1e,f; the size of ZnO–SnO_x_ (5 mM) ranged from 15 to 65 nm (average diameter 32.3 ± 1.8 nm) and that of ZnO–SnO_x_ (10 mM) was 12–78 nm (average diameter 32.8 ± 2.1 nm). A higher concentration of SnO_x_ precursor did not cause a notable increase in coating thickness.

#### 2.1.2. Colloidal Suspension and Stability

The hydrodynamic sizes and zeta potentials of the core–shell nanoparticles, determined by dynamic light scattering measurements, are given in Table 1. The size distribution of bare ZnO lay in the range of 30–100 nm, with a mean diameter of ~68 nm. After SnO_x_ coating, the hydrodynamic size of ZnO–SnO_x_ increased significantly, and the size distribution became broader; for ZnO–SnO_x_ (5 mM), the size varied from 30 nm to 700 nm (mean diameter ~230 nm) and ZnO–SnO_x_ (10 mM) 30–740 nm (mean diameter ~250 nm). The increase in hydrodynamic size for core–shell nanoparticles was due to particle agglomeration, as there was no capping agent on the SnO_x_ layer. This is also reflected in the zeta potential results for ZnO and ZnO–SnO_x_ nanoparticles. As shown in Table 1, the ZnO nanoparticles gave a zeta potential of about 42.54 mV in the presence of the surface ligand of (3-aminopropyl)trimethoxysilane (APTMS), which indicates its high colloidal stability [33]. In the cases of the ZnO–SnO_x_ (5 mM) and ZnO–SnO_x_ (10 mM) particles, the zeta potential values decreased to 14.48 mV and 7.20 mV, respectively, due to the lack of a surface functional group on the SnO_x_ coating. Therefore, the colloidal stability was lowered, and agglomeration of nanoparticles occurred.

#### 2.1.3. Spectroscopic Analysis

**Figure 2:**
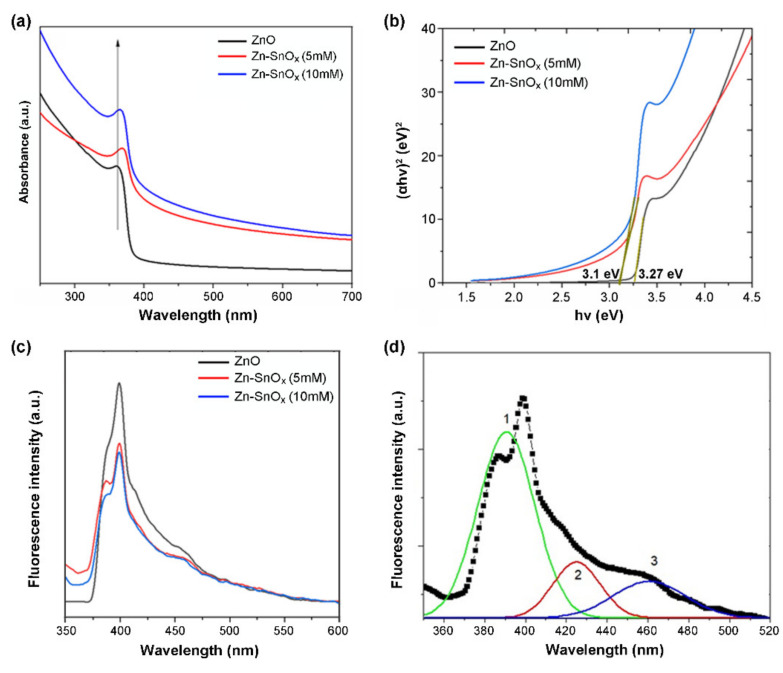
(**a**) UV–visible absorbance, (**b**) Tauc plot, (**c**) PL spectra of ZnO and ZnO–SnO_x_ core–shell nanoparticles, and (**d**) Gaussian fitting curves (1, 2, and 3) represent the deconvolution result of the PL spectrum of the ZnO/SnO_x_ (5 mM) sample.

The UV–Vis absorption spectrum of ZnO nanoparticles shows a sharp absorption edge at 362 nm, which can be assigned to the intrinsic band gap absorption of ZnO caused by electron transitions from the valence band to the conduction band (Figure 2a) [34]. For SnO_x_-coated ZnO using two different concentrations of precursor, i.e., 5 mM and 10 mM SnCl_2_·2H_2_O, slight blue shifts of the absorption band to 366 nm for ZnO–SnO_x_ (5 mM) and to 370 nm for ZnO–SnO_x_ (10 mM) were observed. This could be attributed to the strong electronic coupling between ZnO and SnO_x_ [35]. Since the light absorbance property is directly related to the bandgap of the material [36], the bandgaps of the ZnO and ZnO–SnO_x_ nanoparticles were calculated from the Tauc plot of (αhν)^2^ versus photo energy (hν) in Figure 2b, as all the materials in this core–shell structure are direct bandgap semiconductors [37]. As shown in Figure 2b, the bandgap of the photocatalysts was slightly reduced, from 3.27 eV for ZnO to 3.1 eV after coating SnO_x_ onto ZnO.

The photoluminescence (PL) spectra of the samples were measured to assess the transfer, separation, and recombination behavior of photo-generated electron and hole pairs (Figure 2c). In general, a lower PL intensity causes reduced exciton recombination, leading to a higher photocatalytic activity [38,39]. In the case of ZnO–SnO_x_ core–shell nanoparticles, the intensity of the PL spectrum was significantly quenched compared to the ZnO nanoparticles. Three emission peaks were found after deconvolution with the corresponding Gaussian fitting (Figure 2d), i.e., a near-band-edge UV emission (curve 1) and a broad defect-related visible emission (curves 2 and 3).

### 2.2. Enhancement in Chemical Stability of ZnO–SnO_x_ Core–Shell Nanoparticles

**Figure 3:**
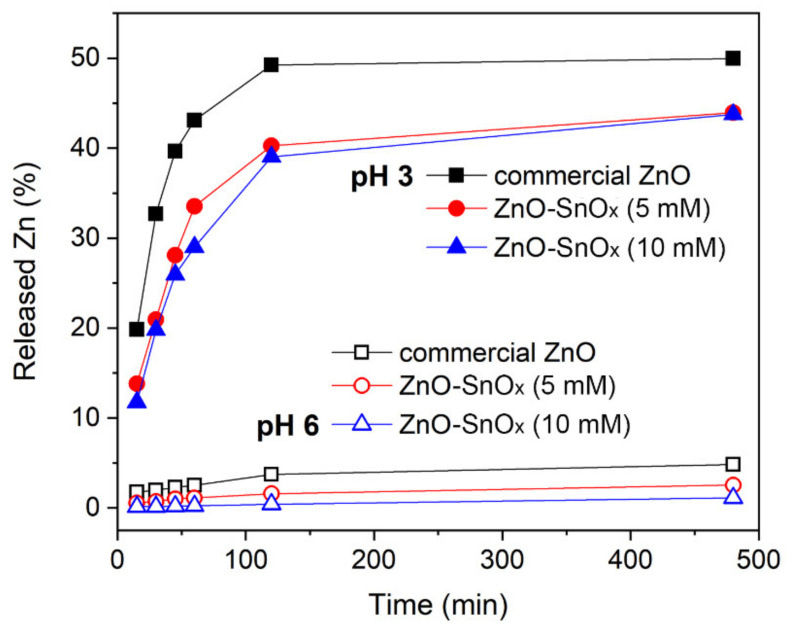
Released zinc ions from ZnO and ZnO–SnOx core–shell nanoparticles at pH 6.0 and pH 3.0 with the lapse of time of 6 h.

The chemical resistance of the core–shell ZnO–SnO_x_ and ZnO nanoparticles was tested in acidic solution, at pH 6.0 and 3.0. The dissolution of ZnO is presented in percentage of released Zn^2+^ ions from the total amount of zinc in commercial ZnO, ZnO–SnO_x_ (5 mM), and ZnO–SnO_x_ (10 mM) nanoparticles at pH 6.0 and 3.0, and this is summarized in Figure 3. At pH 6.0, it was found that the dissolution of ZnO particles after 1 h was about 2.5%, which is higher than that of the core–shell nanoparticles, i.e., 1.1% for ZnO–SnO_x_ (5 mM) and only 0.2% for ZnO–SnO_x_ (10 mM). However, at pH 3.0, the dissolution after 1 h was 43%, 33% and 29%, respectively, for the three nanomaterials. After 2 h, the dissolution of zinc at both pH 6.0 and 3.0 almost reached the maximum. At pH 6.0, the dissolution of zinc was about 3.7%, 1.6% and 0.4% for the ZnO, ZnO–SnO_x_ (5 mM), and ZnO–SnO_x_ (10 mM) nanoparticles, respectively. At pH 3.0, the dissolution was 50% for ZnO and 40% for ZnO–SnO_x_. Tin oxide (SnO_2_) is known for the advantages of having high stability in acidic and basic solutions and in oxidizing environments at higher temperatures [40]. Our results show that, although it was more chemically resistant, the SnO_x_ coating in this work had amorphous nature, and therefore acid diffusion was stronger at a lower pH, and the dissolution of the ZnO was higher. However, compared to the non-coated nanoparticles, the resistance of ZnO nanoparticles with a SnO_x_ coating to acidity was enhanced.

### 2.3. Physical–Chemical Properties of Hybrid Nanocomposite Coatings

#### 2.3.1. Crosslinking of Chitosan with GA

**Figure 4:**
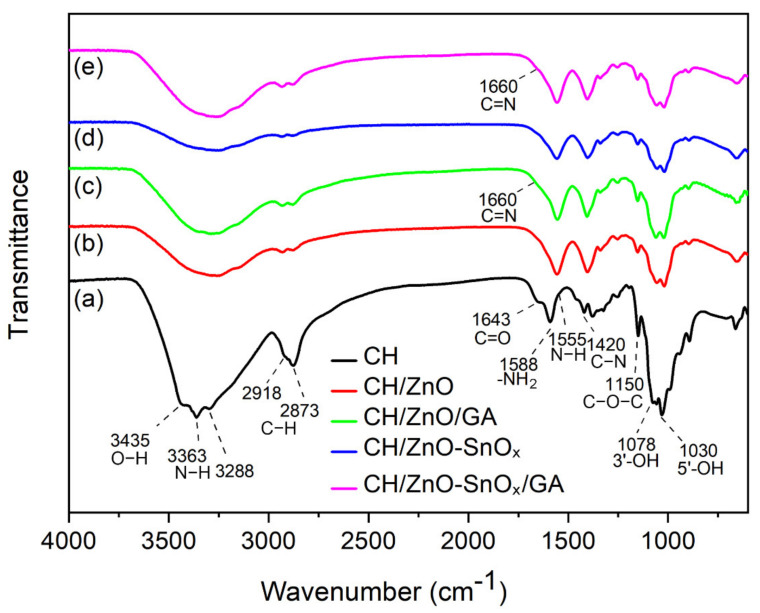
FTIR spectra of (**a**) chitosan, and chitosan nanocomposites of (**b**) chitosan/ZnO, (**c**) CH/ZnO/GA, (**d**) CH/ZnO–SnO_x_, and (**e**) CH/ZnO–SnO_x_/GA.

The FTIR spectra of chitosan and chitosan nanocomposites, both non-crosslinked and crosslinked, are shown in Figure 4. For the chitosan coating (curve a), the characteristic band centered at 3435 cm^−1^ can be attributed to the O-H stretching vibration, while the bands centered at 3363 and 3288 cm^−1^ originated from the absorption of N-H stretching vibrations originating from the non-acetylated 2-aminoglucose primary amine (-NH_2_) in the chitosan chain. The absorption at 2918 and 2873 cm^−1^ corresponds to the axial stretching vibration of C-H bonds in the CH_3_ group at the end of the chitosan chain. The absorption bands centered at 1643, 1555, and 1420 cm^−1^ can be assigned to the stretching vibration of C = O (amide I), the bending vibration of N-H (amide II), and the stretching vibration of C-N (amide III) in the amide group of the residual *N*-acetyl side group, respectively. Additionally, the band centered at 1588 cm^−1^ is from the bending vibration of the primary amine (-NH_2_) from chitosan. Some other functional moieties in the polysaccharide ring or the end group of chitosan also exhibit characteristic absorption bands, such as the band centered at 1150 cm^−1^ for the asymmetric stretching vibration of C-O-C, and at 1078 and 1030 cm^−1^ for the stretching vibration of C-OH groups at the C3 and C5 positions in the polysaccharide ring of chitosan. Upon loading the nanoparticles of ZnO (curve b and c) or ZnO–SnO_x_ (curve d and e), the bands of O-H and N-H stretching vibration shifted towards lower frequencies (ranging from 3370 to 3155 cm^−1^) compared to chitosan. It was also observed that the vibration of the amide group is much compressed. The bending vibration of primary amine (-NH_2_) shifted to a lower frequency at around 1557 cm^−1^, which implies an interaction between nanoparticles and the chitosan chain. For crosslinked chitosan–nanoparticles composites, a small increase in intensity at 1660 cm^−1^ manifests in curves c and e, suggesting a newly formed imine group (C=N) in the Schiff base after the crosslinking of chitosan.

#### 2.3.2. Thermal Stability

**Figure 5:**
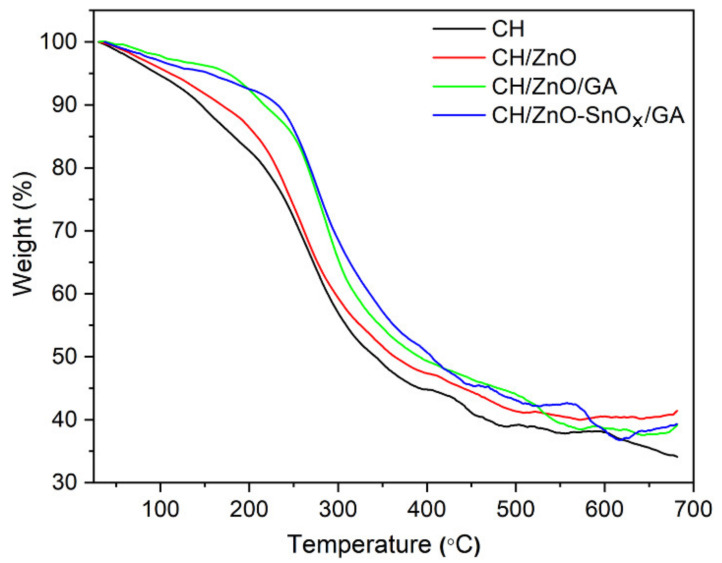
TGA curves of the prepared chitosan and chitosan nanocomposite coatings.

The thermal stability values of the nanocomposites (crosslinked and non-crosslinked) are presented in Figure 5. A three-step weight loss can be observed for all the samples. The first weight loss occurred at temperatures below 100 °C, attributed to moisture loss (5–9%). The second weight loss step, between 140 °C and 220 °C, occurred due to the loss of crystalline/trapped water and the initial decomposition of chitosan (8–11%), whereas the third and major weight loss of 25–40%, in the temperature range of 220–300 °C, was mainly due to the decomposition of the functional group and the polymer backbone. For thermal treatments higher than 300 °C, a much slower rate of weight loss was observed, which corresponds to the continuation of decomposition and the carbonization of the polymer. From the thermogravimetric analysis, it is clear that the thermal stability of the developed nanocomposites was improved upon loading with ZnO nanoparticles. Similar trends were reported in previous studies [41]. A further enhancement of thermal stability was achieved upon crosslinking chitosan with GA, whereby the weight loss decreased from 28% for chitosan–ZnO to 14% for chitosan–ZnO–GA at 250 °C. Among all the compositions, GA-crosslinked chitosan containing ZnO–SnO_x_ (5 mM) core–shell nanoparticles had the highest thermal stability. Thus, the crosslinking of chitosan and the incorporation of ZnO–SnO_x_ (5 mM) core–shell nanoparticles could lead to an improvement in the thermal stability of chitosan films/coatings. Additionally, the moisture loss in GA crosslinked chitosan nanocomposites was lower (3–4%) than that in chitosan coatings (7–9%), indicating a ca. 50% improvement in water uptake with crosslinking [12].

### 2.4. Effect of Crosslinking of Chitosan and Incorporation of Nanoparticles on Water Uptake

The water contact angle (WCA) reflects the relative hydrophobicity or hydrophilicity of a surface and corresponds to its wettability. A higher contact angle indicates lower wettability, and vice versa. The values of the WCA and swelling ratio of the chitosan and crosslinked chitosan coatings are listed in Table 2. The WCA of the glass surface without coating was about 32.6°, whereas the WCA of chitosan- (CH 1%) and crosslinked chitosan- (CH 1%/GA 2.5%) coated glass surfaces increased to 55.5° and 62.5°, respectively.

The WCA of chitosan–ZnO increased to 57.9°, 64.5°, and 75.5° with ZnO incorporation from 1% to 10%, and the swelling ratio was correspondingly reduced (see Table S1 in Supplementary Materials). Increased amounts of ZnO nanoparticles lead to a reduction in the tendency of the coating to absorb water, and thus WCA increases [12,17]. A similar trend was also observed for chitosan–ZnO–SnO_x_ (5 mM) samples, and the swelling ratios were similar to those for chitosan coatings containing ZnO nanoparticles. In the case of GA-crosslinked chitosan–ZnO and chitosan–ZnO–SnO_x_ (5 mM) coatings, the WCA values were higher than those of their counterparts without GA crosslinking, and the values of WCA also increased with the increasing percentage of involved nanoparticles.

### 2.5. Photocatalytic Performance of ZnO–SnO_x_ Core–Shell Nanoparticles under Visible Light Irradiation

**Figure 6:**
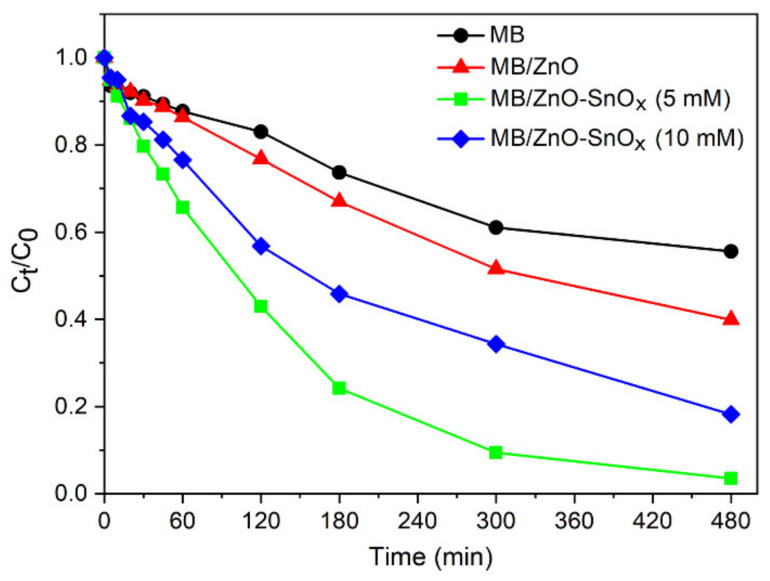
Degradation kinetics of methylene blue (MB) dye (20 µM aqueous solution) mixed with ZnO, ZnO–SnO_x_ (5 mM) and ZnO–SnO_x_ (10 mM) nanoparticles under visible light irradiation, where C_0_ and C_t_ are the concentrations at the beginning and at a certain time of irradiation, respectively.

Methylene blue (MB), a redox-active agent, has a tendency to accept electrons upon being exposed to a reducing agent, and be degraded into its reduced form, leucomethylene blue (LMB), a colorless compound [42,43]. Taking advantage of this feature, the photocatalytic effects of ZnO–SnO_x_ were evaluated through the photodegradation of MB in contact with photocatalysts. Figure 6 presents the degradation kinetics (C_t_/C_0_) of MB, wherein the concentration of remaining MB at different light irradiation times was calculated based on the optical absorbance. It was found that around 40% MB was degraded under visible light irradiation after 8 h without any photocatalysts (photolysis). In the presence of ZnO nanoparticles, the degradation of MB increased to 58%. Compared to ZnO, ZnO–SnO_x_ (5 mM) core–shell nanoparticles dramatically enhanced the rate of degradation, and 98% of the MB was degraded. When the coating thickness was increased, as was the case for ZnO–SnO_x_ (10 mM) core–shell nanoparticles, the rate of MB degradation was lower than that of ZnO–SnO_x_ (5 mM). There are many studies on chitosan-based semiconductor photocatalysts, which have shown the ability to immobilize the catalyst in chitosan for the effective separation and reuse of the catalyst [44]. Chitosan itself is not a photo-absorber or a semiconductor. Due to the electrostatic or chelating interaction between chitosan and dye or organic waste, chitosan exhibits a high adsorption capacity. It helps bridge the dye molecule and photocatalyst [45], but is not directly involved in the photocatalysis process. Therefore, the use of chitosan in a matrix with a ZnO–SnO_x_ photocatalyst has not been studied in this work for its photocatalytic properties. However, it can be expected that with a chitosan matrix, the photocatalytic performance of the ZnO–SnO_x_ nanoparticles will be slightly hampered due to the loss of surface area for contacting the MB.

### 2.6. Antifouling Activity of Chitosan Nanocomposite Coatings

**Figure 7:**
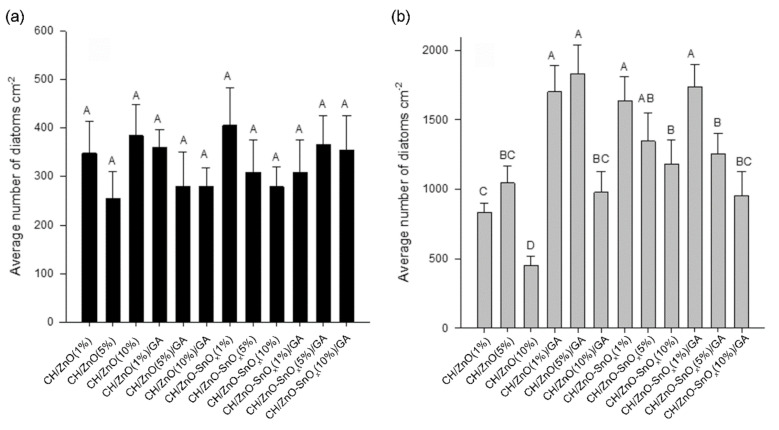
The average densities of diatoms on the tested nanocomposite coatings under (**a**) dark conditions and (**b**) light conditions. The data are presented as the average number (*n* = 3) of diatoms per cm^2^ plus standard deviation. Significant differences between means according to the Tukey’s test (*p* < 0.05) are indicated by different letters above the bars. Different superscript letters represent the multiple comparison results, that differs significantly (i.e., A is significantly higher than the others; D is significantly lower than the others). The superscript of AB or BC indicated that the results are not significantly different from A, B or C.

After 4 days in the dark, well-developed biofilms dominated by diatoms (Bacillariophyceae) were formed on the surfaces of all tested coatings. The most common diatom genera found in these biofilms were *Navicula*, *Cocconeis*, and *Amphora*. Previous studies have suggested that these genera of diatoms were frequently found on antifouling coatings [46,47]. Since no apparent differences (ANOVA, HSD, *p* > 0.05) were exhibited between the densities of the diatoms on the tested coatings in the dark (Figure 7a), we can assume that neither the physical properties (such as WCA) nor the chemical compositions of different coatings affect the antifouling performance of coatings in the dark.

Under natural light, *Navicula* was the most common genera of diatoms found on all the tested coatings. The densities of these diatoms were around five times higher than those from the experiments carried out in the dark (Figure 7b). Meanwhile, significant differences (ANOVA, HSD, *p* < 0.05) were found between the densities of diatoms on different nanocomposite coatings, which is different to the results from the dark experiment. The lowest densities of diatoms were observed on the CH (1%)/ZnO (10%) coating.

## 3. Discussion

The light absorption characteristics of a photocatalyst are among the most critical properties determining its photocatalytic activity. The observed reduction in bandgap in Figure 2b for ZnO–SnO_x_ compared to ZnO can be correlated to the rich donor defects providing additional deep donor levels between the valence and the conduction band [36,48]. The deconvoluted PL spectrum of ZnO–SnO_x_ in Figure 2d exhibits three peaks. The emission at around 390 nm is from the near-band-edge transition of ZnO [38,49]. In addition, the emission of surface-localized excitons on the SnO_x_ shell layer is also in this wavelength range [27]. The visible emissions at 425 nm and 460 nm are thought to arise from defect-related states such as oxygen vacancies generated during the formation of ZnO nanoparticles [50].

**Figure 8:**
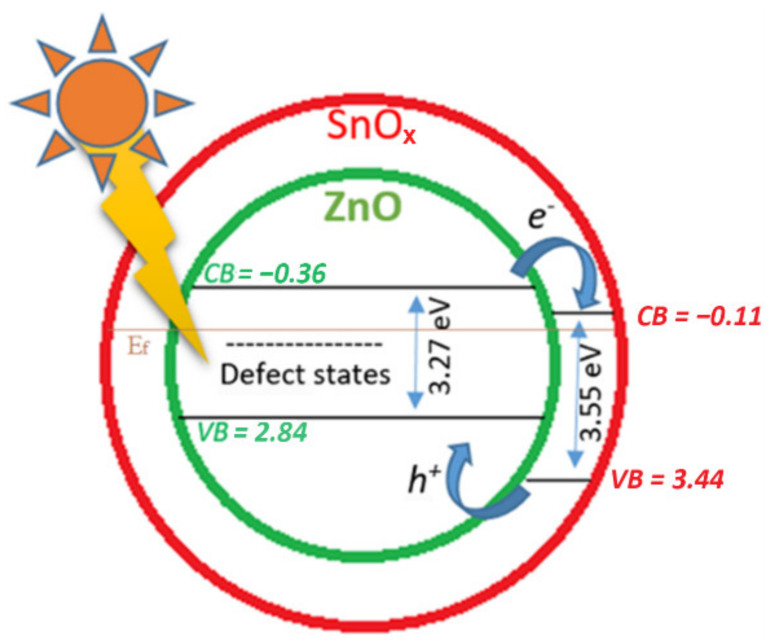
Schematic diagram of the photocatalytic mechanism of the ZnO–SnO_x_ core–shell structure under visible light irradiation, where the values of the lowest conduction band (CB) and the highest valence band (VB) of ZnO and SnO_x_ are relevant to the Fermi energy (E_f_).

A schematic diagram representing the charge–transfer processes for ZnO–SnO_x_ is illustrated in Figure 8. For ZnO–SnO_x_ heterojunction nanoparticles, the photogeneration of electron–hole pairs and their recombination define the photoactivity [51]. Both ZnO and SnO_x_ are n-type semiconductors, and SnO_x_ is a better electron acceptor than ZnO because the conduction band potential of SnO_x_ is more positive than that of ZnO. By coupling a larger-bandgap material (amorphous tin oxide bandgap: 3.6 eV) to a smaller-bandgap semiconductor (ZnO bandgap: 3.3 eV), a type-II heterostructure is formed. Following the photo-generation of electron–hole pairs, the electrons can move from the conduction band (CB) of ZnO to the CB of SnO_x_, and the holes can move from the valence band (VB) of SnO_x_ to the VB of ZnO. Attributed to the transparency of the thin layer coating of SnO_x_, visible light can reach ZnO nanoparticles easily (Figure 8), and electrons can be activated by light illumination (shown in Figure 2c). Coupling a SnO_x_ coating with a ZnO core could promote the charge transfer efficiency and the spatial separation of the photogenerated carriers. Thus, the ZnO–SnO_x_ core–shell nanoparticles are expected to show better photocatalytic efficiency than the ZnO nanoparticles [52], as well as a higher chemical stability, as previously shown in Section 2.2.

The FTIR analysis indicates that upon loading ZnO or ZnO–SnO_x_ onto chitosan, the stretching vibration of O-H and N-H shifts to a lower frequency. This could be due to the formation of intermolecular hydrogen bonds between the nanoparticles and chitosan.

In the WCA study, the chitosan coating showed higher hydrophobicity than the surface of the microscope glass slide, where silanol groups (Si-OH) are present at a high density, which was also found in previous work [53]. This is associated to the hydrophobic acetyl groups and unprotonated amine groups present in the chitosan chain. The wettability of the chitosan coating is decreased upon crosslinking due to the increase in hydrophobicity of the coating, which was confirmed by the reduction in the swelling ratio, i.e., water uptake, of the coatings from 0.56% to 0.22%. This is attributed to the reduction in the number of amine groups and the increased cohesiveness of the surface following the crosslinking of chitosan [54,55,56]. This also applies to the crosslinked chitosan–nanoparticles composite, wherein some of the amine groups crosslinked with the aldehyde groups of GA to form azomethine units, leading to a lower number of available amino groups in chitosan for hydrogen bonding with water, and thus the WCA increased.

The enhanced degradation kinetics of MB using ZnO–SnO_x_ (5 mM) compared to ZnO could be attributed to the efficient electron transfer taking place in the ZnO–SnO_x_ photocatalyst, as discussed earlier. When the coating thickness of SnO_x_ was increased, decreased degradation kinetics were observed for ZnO–SnO_x_ (10 mM). This might be due to the higher amount of available surface defects in the case of ZnO–SnO_x_ (5 mM) with a very thin SnO_x_ coating compared to ZnO–SnO_x_ (10 mM) with a thick SnO_x_ coating.

In the current study, the lowest densities of diatoms were observed on the CH (1%)/ZnO (10%) coating. This result is similar to that in our previous study, wherein we demonstrated that a chitosan–ZnO nanocomposite coating had high anti-diatom activity [12], attributed mainly to the reactive oxygen species (ROS) released by ZnO during the photocatalysis process, and we also determined that the presence of active amine groups is responsible for the antimicrobial activity of chitosan [7,57]. The antifouling performance of chitosan nanocomposites crosslinked with the highest ZnO concentrations, i.e., CH (1%)/ZnO (10%)/GA (2.5%) and CH (1%)/ZnO–SnO_x_ (10%)/GA (2.5%), was as good as that of the CH (1%)/ZnO (1%) and CH (1%)/ZnO (5%) nanocomposite coatings. This result indicates that the photocatalytic activity of the greater amount of ZnO or ZnO–SnO_x_ could compensate for the weakened antifouling property due to crosslinking, and therefore enhance the antifouling activity of the nanocomposites. Although the antifouling activity of CH (1%)/ZnO–SnO_x_ (10%)/GA (2.5%) was slightly lower than that of the CH (1%)/ZnO (10%) nanocomposite coating, considering its enhanced thermal and chemical stability and lower water uptake, the active life of the CH (1%)/ZnO–SnO_x_ (10%)/GA (2.5%) coating is expected to be much extended.

## 4. Materials and Methods

### 4.1. Materials

Chitosan with a molecular weight of 100–300 kDa was obtained from Acros Organics, USA. Aqueous suspensions of ZnO nanoparticles (50 wt.% in H_2_O, <100 nm sizes), glacial acetic acid, tin(II) chloride dehydrate (SnCl_2_·2H_2_O), glutaraldehyde (Grade II, 50 wt.% in H_2_O) and MB solution (0.05 wt.% in H_2_O) were purchased from Sigma-Aldrich, St. Louis, MO, USA.

### 4.2. Hydrothermal Synthesis and Characterization of ZnO–SnO_x_ Core–Shell Nanoparticles

ZnO–SnO_x_ core–shell nanoparticles were synthesized by a hydrothermal process modified from the method reported by Wang et al. [35]. Typically, 0.568 mL of commercial ZnO colloidal nano-suspension (50 wt.%) is gradually mixed with 80 mL ethanol solution containing 0.090 g (5 mM) of SnCl_2_·2H_2_O. The solution is then transferred into a 100 mL Teflon-lined autoclave and the hydrothermal process is performed at 120 °C for 16 h. The obtained white precipitate is washed several times with de-ionized water, dried at 70 °C for 5 h, and then calcined for 1.5 h to obtain a white powder of ZnO–SnO_x_ core–shell nanostructures. The calcination of ZnO is carried out at 250 °C to generate the highest amount of surface defects, as shown in one of our previous studies [38]. For comparison, twice the amount of SnCl_2_·2H_2_O, i.e., 10 mM, is used to synthesize the ZnO–SnO_x_ core–shell nanostructures. Finally, both the obtained samples of ZnO–SnO_x_ (5 mM) and ZnO–SnO_x_ (10 mM) are stored in an airtight glass container under ambient conditions for further analysis and use.

The particle size distribution and surface charge of the prepared core–shell nanoparticles were determined via dynamic light scattering and zeta potential measurements using Delsa Nano C (Beckman Coulter Inc., Brea, CA, USA). The photoluminescence (PL) and optical absorption spectra were recorded using a fluorescence spectrometer (LS 55, PerkinElmer Inc., Waltham, MA, USA) and UV–visible–NIR spectrometer (Lambda-750, PerkinElmer Inc., Waltham, MA, USA), respectively. The morphology of the particles was further characterized via transmission electron microscopy (TEM, JEM-2100F, JEOL Ltd., Akishima, Tokyo, Japan).

### 4.3. Chemical Stability Study of ZnO–SnO_x_ Core–Shell Nanoparticles

The chemical stability of the ZnO and ZnO–SnO_x_ (5 mM and 10 mM) core–shell nanoparticles was investigated in acidic conditions at pH 3 and 6. For this process, 10 mL of an aqueous suspension of the aforementioned nanoparticles (1 mg/mL) was adjusted to pH 3 or 6 using hydrochloric acid (HCl) and transferred into dialysis bags, which were then placed into a glass beaker containing 1 L of de-ionized water with corresponding pH values for dialysis under magnetic stirring. At a regular interval of 5 min, aliquots of 5 mL of the solution were collected from the glass beaker, and the concentration of leached Zn ions was measured using ICP-OES (inductively coupled plasma–optical emission spectrometry, iCAP 6000 series, Thermo Fisher Scientific Inc., Waltham, MA, USA).

### 4.4. Photocatalytic Performance of ZnO–SnO_x_ Core–Shell Nanoparticles

The photocatalytic efficiencies of ZnO and the synthesized ZnO–SnO_x_ core–shell nanoparticles were examined by studying the degradation of MB under visible light irradiation. In a typical experiment, 3 mg (1 mg/mL) of each type of photocatalyst (ZnO, ZnO–SnO_x_ (5 mM) and ZnO–SnO_x_ (10 mM)) was mixed separately with 30 mL of an aqueous solution of MB (20 μM). A control sample containing only MB (30 mL, 20 μM) was also prepared. All the samples were stirred under dark conditions for 45 min to ensure adsorption and desorption equilibrium prior to the photocatalytic degradation experiments. The photocatalytic degradation of MB was carried out under visible light using a solar simulator (150 W dichroic halogen lamps, Hugo Brennenstuhl GmbH & Co., Tübingen, Germany) from a distance of 10 cm, and it lasted for 8 h. At different time intervals—for example, every 5 or 10 min in the first hour and every hour afterwards—2 mL of aliquots were taken from each sample and centrifuged to remove the particles. The absorbance of the supernatant was measured using a UV–Vis spectrophotometer (Lambda-750, PerkinElmer Inc., Waltham, MA, USA). Accordingly, the concentration of MB that remained in the supernatant was then calculated from the calibration curve of MB, obtained from a UV–Vis measurement of the diluted samples.

### 4.5. Fabrication of Chitosan Nanocomposite Coatings

Firstly, 1% (w/v) of chitosan solution was prepared by dissolving chitosan powder in 1% (v/v) acetic acid. Then, 1, 5 or 10 wt.% (w.r.t. weight of chitosan) of ZnO or ZnO–SnO_x_ (5 mM) nanoparticles was mixed with the chitosan solution under continuous stirring. The obtained chitosan nanocomposite samples were named CH (1%)/ZnO (1/5/10%) or CH (1%)/ZnO–SnO_x_ (1/5/10%), respectively. To decrease water uptake and wettability, glutaraldehyde (GA) was used as a crosslinking agent for the chitosan nanocomposites. Specifically, chitosan samples mixed with ZnO or ZnO–SnO_x_ (5 mM) nanoparticles were mixed with 2.5% (w.r.t. weight of chitosan) of GA and stirred slowly for 6 h for proper crosslinking. The obtained samples were named CH (1%)/ZnO (1/5/10%)/GA (2.5%) or CH (1%)/ZnO–SnO_x_ (1/5/10%)/GA (2.5%), respectively. Two control samples, chitosan and chitosan crosslinked with GA without nanoparticles, were also prepared.

### 4.6. Preparation of Substrates for the Mesocosm Experiment

Glass slides (25 × 75 × 1 mm) were used as a substrate for the coatings. Prior to the experiment, glass slides were cleaned using ethanol in a sonication bath for 15 min, and then washed with DI water and dried. The dry glass slides were dipped in an ethanol solution containing 3 mL of 3-(triethoxysilyl)propylsuccinic anhydride (TESPS; abcr GmbH, Germany), which was then heated until reflux for 6 h followed by drying at room temperature. The glass slides were then uniformly spray-coated with the synthesized chitosan nanocomposite solution and allowed to dry overnight at room temperature (25 °C). Each glass slide was spray-coated for 8 cycles to obtain the appropriate thickness of coating. Glass slides coated with chitosan and chitosan crosslinked with GA were also prepared similarly, and used as controls.

### 4.7. Characterization of Nanocomposite Coating

Surface analysis of the functional groups of the chitosan nanocomposite coatings was performed by Fourier transform infrared spectroscopy (FTIR) (Nicolet iS10, Thermo Fisher Scientific Inc., Waltham, MA, USA) in the range of 4000–600 cm^−1^. Thermo-gravimetric analyses (TGA) of the nanocomposite samples were carried out in a TGA analyzer (Q500, TA instruments, USA) at a heating rate of 10 °C min^−1^ over the temperature range 20–700 °C in a nitrogen atmosphere. To determine the hydrophilicity/hydrophobicity of the nanocomposite coatings, the surface water contact angle (WCA) was determined using a contact angle goniometer (Ossila Ltd., Sheffield, UK), and the WCA was measured at 4–5 different positions on a coated glass slide with different coating compositions. The water uptake property or swelling of the coatings was determined gravimetrically following a previously reported protocol with minor modifications [56]. Specifically, coated glass slides were dried in an oven at 60 °C for 24 h to obtain the initial dry weight (denoted as W_1_). The dried slides were then placed in 60 mL DI water at 30 °C for 48 h under constant agitation for water uptake. The weight of the samples was again measured (wet weight, W_2_) immediately after the excess water was removed by adsorption using Whatman No. 1 filter paper. The percentage of water uptake (swelling) of the coatings was calculated using Equation (1):

% Water uptake value (W_u_) = [(W_2_−W_1_)/W_1_] × 100% (1)

where W_1_ and W_2_ are the weights of the samples in the dry and swollen states, respectively.

### 4.8. Antifouling Activity of Nanocomposite Coatings

The antifouling activities of various chitosan–nanocomposite coatings on glass slides were tested in a mesocosm experiment at the Universidad Catolica del Norte (Coquimbo, Chile). The experiment was conducted in an outdoor 2000 L tank with a flow-through system under light and dark conditions, respectively (Supplementary Materials, Figures S1 and S2). The tank was fed with seawater obtained from La Herradura Bay (29°58′S 71°22′ W) and the water flow was 2 L min^−1^. The temperature of seawater was 13.5 °C and the salinity was 32 ppt. Three independent replicates for each coating were used. The substrates were exposed to biofouling horizontally in a basket at the depth of 5 cm (Figure S1). Two sets of experiments were conducted, one under sunlight (photosynthetically active radiation (pAR) at 400–700 nm with the photosynthetic photon flux density (PPFD) of 1000–1031 µmol m^−2^ s^−1^; UV 35–40 Wm^−2^) and another one in the dark (PAR and UV: 0, Figure S2). After 4 days of exposure, the substrates were collected. The densities of the diatoms on the coatings were counted using a compound light microscope (Zeiss, Germany) under the magnification of 400x, as described in previous reports [58]. The total abundance of diatoms was expressed as the average number of cells per mm^2^. The differences in the densities of diatoms on different nanocomposite coatings were compared using analysis of variance (ANOVA) followed by the Tukey’s honest significant difference (HSD) test. Prior to ANOVA, the normality of the data was verified by the Shapiro–Wilk test. The densities that were significantly different from each other had a threshold of 5%.

## 5. Conclusions

We developed a biopolymer nanocomposite antifouling coating based on a chitosan–ZnO–SnO_x_ nanocomposite. In the preparation of the chitosan nanocomposite, the chemical stability of the photocatalyst ZnO is enhanced by coating a layer of amorphous SnO_x_ shell onto ZnO, which will ensure the long-term application of the photocatalyst. The incorporation of ZnO–SnO_x_ and the crosslinking of chitosan reduced the water uptake of the coatings, and thus the hydrophobicity and swelling properties were improved. The thermal stability was also enhanced owing to the embedding of the nanoparticle, suggesting the increased mechanical strength of the coating, which is beneficial for the application. With improved chemical and thermal stability, as well as hydrophobicity, the feasibility of applying chitosan–ZnO–SnO_x_ nanocomposite coatings for the prevention of marine biofouling has been demonstrated. It has been found that the fouling of the diatom *Navicula* was significantly reduced compared to the uncoated control or chitosan coating samples. Thus, the chitosan matrix with chemically resistant ZnO–SnO_x_ nanoparticles, used as a coating, represents a promising strategy for preventing marine biofouling.

## Figures and Tables

**Table 1:** Hydrodynamic particle diameter and zeta potential values of core–shell nanoparticles.

Particles	Diameter (nm)	Zeta Potential (mV)
Bare ZnO	68.4 ± 0.6	42.54 ± 0.27
ZnO–SnO_x_ (5 mM)	230.6 ± 1.8	14.48 ± 0.34
ZnO–SnO_x_ (10 mM)	250.3 ± 0.9	7.20 ± 0.14

**Table 2:** Water contact angle (WCA) and water uptake values of chitosan and crosslinked chitosan coatings.

Sample	WCA (Degree)	Swelling Ratio (%) ^1^
Glass slides without coating (blank)	32.6 ± 0.7	0
CH (1%)	55.5 ± 1.3	0.56 ± 0.28
CH (1%)/GA (2.5%)	62.5 ± 0.8	0.22 ± 0.06

^1^ Values are given as means ± standard deviation (*n* = 5).

## Data Availability

The data presented in this study are available on request from the corresponding author. The data are not publicly available due to confidentiality issues.

## Supplementary Materials

The following are available online at https://www.mdpi.com/article/10.3390/ijms22094513/s1, Table S1: WCA and swelling ratio of chitosan nanocomposite coatings, Figure S1: A mesocosm under light conditions, Figure S2: A mesocosm experiment under dark conditions.
