# Supplementary material for: Chitosan Nanocomposite Coatings Containing Chemically Resistant ZnO–SnOx Core–shell Nanoparticles for Photocatalytic Antifouling
Source: Int J Mol Sci. 2021 Apr 26;22(9):4513. doi: 10.3390/ijms22094513 (PMC8123458; doi:10.3390/ijms22094513)

Supplementary Materials

Table S1. Water contact angle (WCA) and water uptake values (i.e., swelling ratio) for chitosan nanocomposite coatings.

Sample	WCA (degree)	Swelling ratio (%) [1]
CH (1%)/ZnO (1%)	57.9 ± 1.2	1.22 ± 0.12
CH (1%)/ZnO (5%)	64.5 ± 0.3	1.12 ± 0.41
CH (1%)/ZnO (10%)	75.5 ± 0.9	1.05 ± 0.21
CH (1%)/ZnO-SnO_x (1%)	58.1 ± 1.0	1.28 ± 0.08
CH (1%)/ZnO-SnO_x (5%)	63.4 ± 0.5	1.03 ± 0.14
CH (1%)/ZnO-SnO_x (10%)	72.7 ± 1.2	1.05 ± 0.02
CH (1%)/ZnO (1%)/GA (2.5%)	63.8 ± 0.7	0.89 ± 0.06
CH (1%)/ZnO (5%)/GA (2.5%)	66.8 ± 0.1	0.83 ± 0.09
CH (1%)/ZnO (10%)/GA (2.5%)	78.8 ± 0.4	0.80 ± 0.16
CH (1%)/ZnO-SnO_x (1%)/GA (2.5%)	67.6 ± 1.1	0.73 ± 0.07
CH (1%)/ZnO-SnO_x (5%)/GA (2.5%)	69.3 ± 0.6	0.65 ± 0.18
CH (1%)/ZnO-SnO_x (10%)/GA (2.5%)	77.5 ± 0.3	0.60 ± 0.03

[1] Values are given as means ± standard deviation (n=5).

Figure S1. A mesocosm experiment at the light conditions. The nanocomposite coatings were incubated horizontally in a basket.

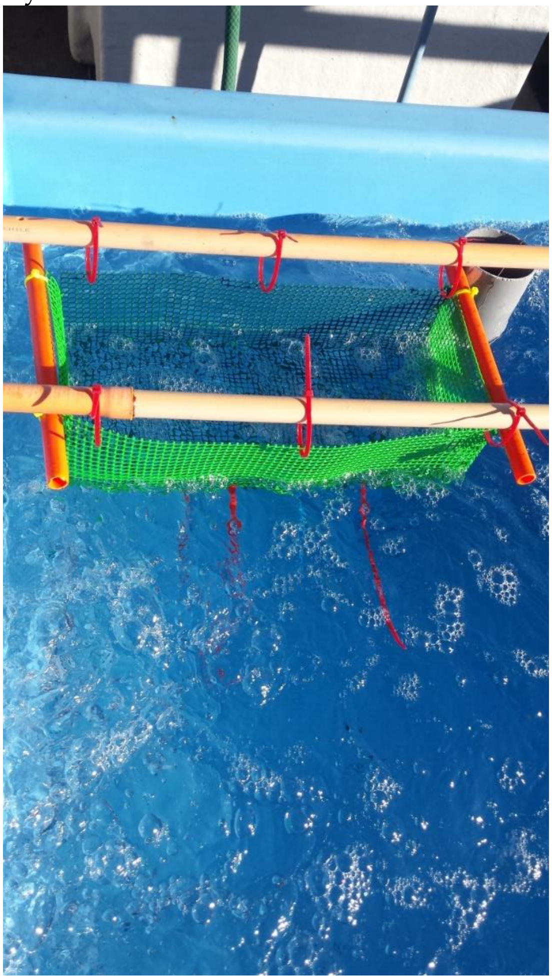

Figure S2. A mesocosm experiment at the dark conditions. In order to prevent the light, the tank was covered with black net.

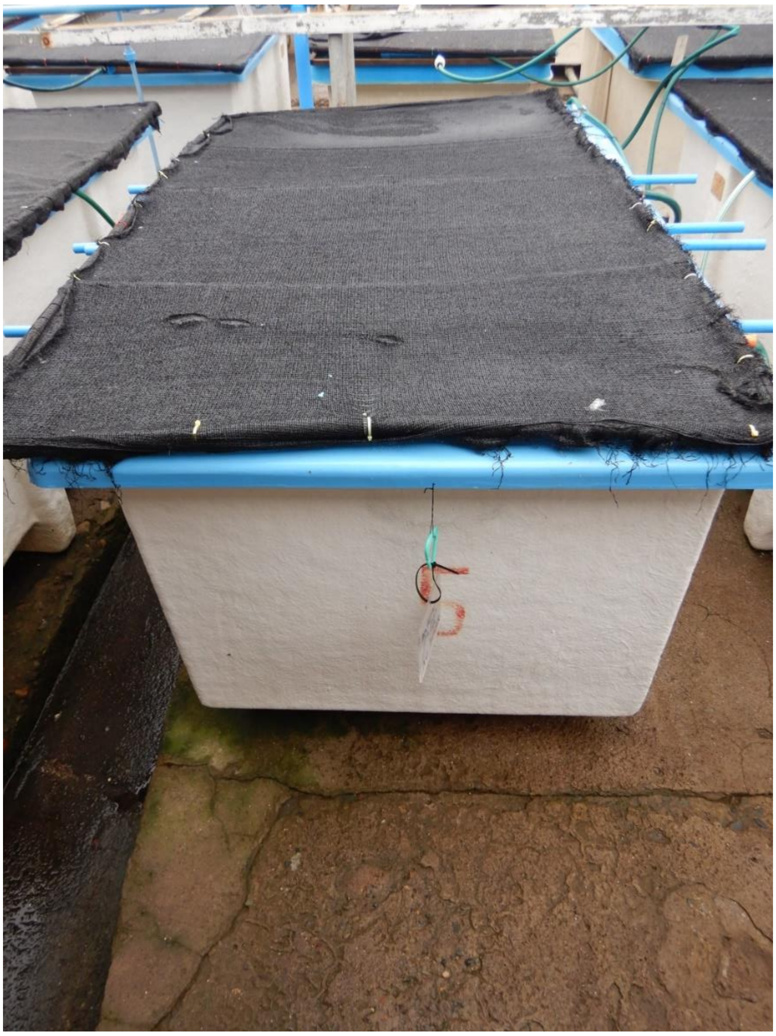

Supplement: Supplementary file 1 [file ijms-22-04513-s001.zip › ijms-1182943-supplementary.pdf]
